# Examining variations in body composition among patients with colorectal cancer according to site and disease stage

**DOI:** 10.1038/s41598-024-61790-0

**Published:** 2024-05-11

**Authors:** Mayra Laryssa da Silva Nascimento, Nithaela Alves Bennemann, Iasmin Matias de Sousa, Mara Rubia de Oliveira Bezerra, Gabriela Villaça Chaves, Sara Maria Moreira Lima Verde, Silvia Fernandes Maurício, José Barreto Campello Carvalheira, Maria Carolina Santos Mendes, Ana Lucia Miranda, Jarson Pedro da Costa Pereira, M. Cristina Gonzalez, Carla M. Prado, Ana Paula Trussardi Fayh

**Affiliations:** 1 PesqClin Lab, Onofre Lopes University Hospital, Brazilian Company of Hospital Services (EBSERH), Federal University of Rio Grande do Norte, Natal, RN Brazil; 2 Health Sciences Center, Federal University of Rio Grande do Norte, Avenida Senador Salgado Filho, No 3000, Natal, RN 59078-970 Brazil; 3 National Institute of Cancer, Rio de Janeiro, RJ Brazil; 4 State University of Ceará, Fortaleza, CE Brazil; 5 Federal University of Ouro Preto, Ouro Preto, MG Brazil; 6 Division of Oncology, Department of Anesthesiology, Oncology and Radiology, School of Medical Sciences, State University of Campinas (UNICAMP), Campinas, SP Brazil; 7 Liga Norteriograndense Contra o Câncer, Natal, Rio Grande do Norte Brazil; 8 Department of Nutrition, Federal University of Pernambuco, Recife, PE Brazil; 9 Federal University of Pelotas, Pelotas, RS Brazil; 10 Human Nutrition Research Unit, Department of Agricultural, Food and Nutritional Science, University of Alberta, Edmonton, AB Canada

**Keywords:** Metastasis, Oncology, Epidemiology

## Abstract

Patients with colorectal cancer (CRC) often exhibit changes in body composition (BC) which are associated with poorer clinical outcomes. Many studies group colon and rectal cancers together, irrespective of staging, potentially affecting assessment and treatment strategies. Our study aimed to compare BC in patients with CRC focusing on tumor location and metastasis presence. A total of 635 individuals were evaluated, with a mean age of 61.8 ± 12.4 years and 50.2% female. The majority had rectal cancer as the primary cancer site (51.0%), and 23.6% had metastatic disease. The first regression model showed tumor site and metastasis as independent factors influencing skeletal muscle (SM), skeletal muscle index (SMI), and visceral adipose tissue variability (all *p* values < 0.05). The second model, adjusted for BMI, indicated tumor site as the primary factor affecting SMI variations (adjusted R^2^ = 0.50 *p* < 0.001), with colon tumors inversely associated with SM (standardized β − 2.15(− 3.3; − 0.9) *p* < 0.001). A third model, considering all the confounders from the directed acyclic graphs, was constructed and the found association remained independent. Our findings highlight significant BC variations in patients with CRC, influenced by tumor location and metastases presence, underscoring the need for location-specific assessment in CRC management.

## Introduction

Cancer remains a major cause of morbidity and mortality worldwide, affecting all levels of human development or economic status. In 2020, there were approximately 19.3 million new cancer cases and nearly 10 million cancer-related deaths^1^. Individuals with cancer experience considerable metabolic changes, including common nutritional issues such as malnutrition, anorexia, cachexia, and sarcopenia. The severity of these changes often varies based on the tumor’s location and stage and is further influenced by anticancer treatments^2–4^. Additionally, excess adipose tissue, a known risk factor for cancer, also impacts prognosis and treatment^5,6^. Consequently, the assessment of body composition (BC) in patients with cancer is crucial. It not only contributes to diagnosing related conditions but also aiding effective clinical management of these individuals^5,7,8^.

Colorectal cancer (CRC) is notably the third leading cause of cancer mortality globally, accounting for over 1.85 million cases and 850,000 deaths annually^1,9^. Anatomically, the colon (the larger part of the large intestine), and the rectum, (the terminal part of the intestine) differ in location, blood supply, drainage, and innervation. These differences influence the primary tumor’s invasive growth, as well as the surgical approach and treatment strategies^9,10^. However, most studies evaluating BC often group colon and rectum cancers together, potentially impacting treatment and nutritional intervention for individual patients^11^.

In cases of metastases, where cancer cells spread via blood or lymphatic systems, patients undergo metabolic changes, including increased inflammation, which can directly affect their nutritional status^12,13^. In advanced CRC, muscle loss often occurs concomitantly with weight and adipose tissue losses. However, muscle loss can be subtle in stages I–III, occurring independently of weight loss or adiposity reduction, making its early detection crucial for tailored therapeutic intervention^14^. It is important to highlight the significant impact of inflammation in metastatic cancer, particularly how metastases restructure local tissue, partly by recruiting immune/inflammatory cells, among other mechanisms^15^.

The different approaches and characteristics observed in CRC, depending on the primary tumor’s site and the presence or absence of metastases, underscore the need to differentiate these patients’ groups when analyzing body tissue distribution. Despite its importance, few studies have compared the BC of patients with CRC, with these considerations in mind. Therefore, this study aims to compare the BC of patients with CRC, specifically considering the site of the primary tumor (colon or rectum) and the presence or absence of metastases.

## Results

**Figure 1:**
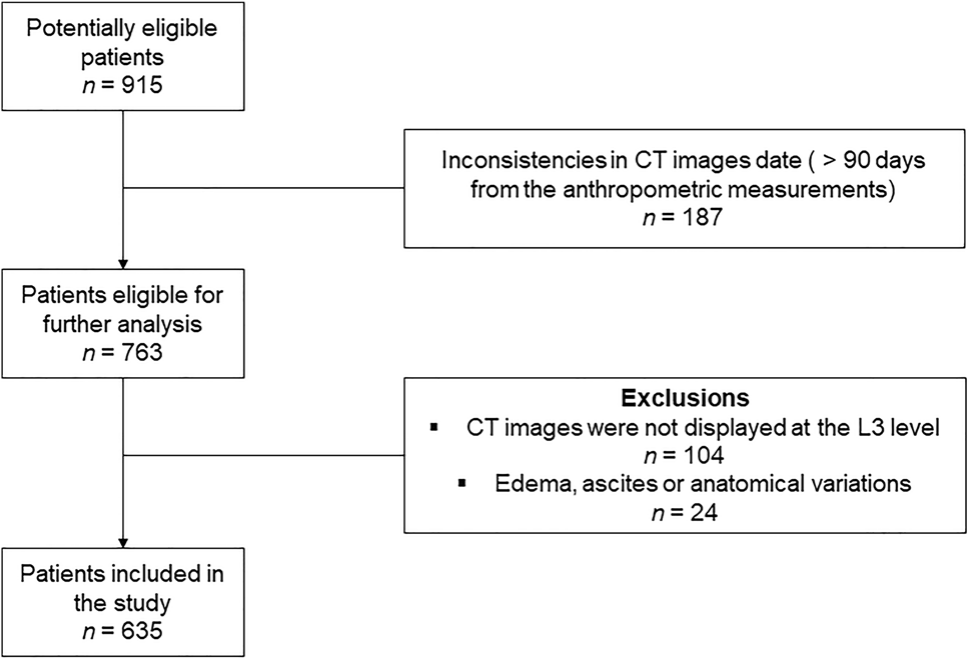
Study flowchart. CT, computed tomography.

A total of 915 patients were screened for eligibility, 152 had CT image taken > 90 days of the anthropometric evaluation, 104 did not have CT image including the L3, 24 had edema, ascites or anatomical variations that could influence in the analysis of BC by CT. Therefore, 635 patients were included in the analysis (Fig. 1). Of these, 49.0% had colon cancer, 51.0% had rectal cancer, and 23.6% had metastasis. Regarding sociodemographic data, more than half of the patients were older adults, with a similar distribution between males (49.8%) and females (50.2%), and the majority (51.3%) was non-Caucasian. Table 1 presents the sociodemographic and clinical characteristics of patients with CRC according to tumor site and the presence of metastasis. When comparing groups according to tumor location, patients with rectal cancer were older, more frequently non-Caucasian, and had a lower frequency of metastasis compared to patients with colon cancer.

**Table 1:** Sociodemographic and clinical characteristics of patients with colon and rectal cancer according to tumor location and presence of metastasis (*N* = 635).

Characteristics	Total (n = 635)	Colon (n = 311)	Rectum (n = 324)	*p*	Without metastasis (n = 485)	With metastasis (n = 150)	*p*
Sociodemographic							
Age (years)	61.8 ± 12.4	60.7 ± 12.3	62.7 ± 12.5	**0.047**^a^	62.1 ± 12.3	60.7 ± 12.8	0.227^a^
Sex				0.550^b^			0.496^b^
Men	316 (49.8%)	151 (48.6%)	165 (50.9%)		245 (50.5%)	71 (47.3%)	
Women	319 (50.2%)	160 (51.4%)	159 (49.1%)		240 (49.5%)	79 (52.7%)	
Ethnicity^1^				< **0.001**^b^			0.644^b^
Caucasian	307 (48.7%)	175 (57.8%)	132 (41.4%)		231 (48.8%)	76 (51.%)	
Non-caucasian	315 (51.3%)	128 (42.2%)	187 (58.6%)		242 (51.2%)	73 (49.0%)	
Smoking^2^				< **0.001**^b^			0.236^b^
Yes/Former smoker	195 (38.8%)	68 (28.3%)	127 (48.3%)		154 (40.2%)	79 (65.8%)	
No	308 (61.2%)	172 (71.7%)	136 (51.7%)		229 (59.8%)	41 (34.2%)	
Alcohol intake^3^				0.551^b^			0.515^b^
Yes/Former drinker	187 (36.0%)	100 (37.2%)	87 (34.7%)		139 (35.2%)	48 (38.4%)	
No	333 (64.0%)	169 (62.8%)	164 (65.3%)		256 (64.8%)	77 (61.6%)	
Marriage status^4^				0.882^b^			0.952^b^
With partner	270 (61.6%)	145 (62.0%)	125 (61.3%)		208 (61.7%)	62 (61.4%)	
Without partner	168 (38.4%)	89 (38.0%)	79 (38.7%)		129 (38.3%)	39 (38.6%)	
Educational level^5^				0.198^b^			0.773^b^
< 8 years of education	234 (57.1%)	122 (54.2%)	112 (60.5%)		181 (57.5%)	53 (55.8%)	
≥ 8 years of education	176 (42.9%)	103 (45.8%)	73 (39.5%)		134 (42.5%)	42 (44.2%)	
Clinical							
Type of cancer							< **0.001**^b^
Colon	311 (49.0%)	–	–	–	216 (44.5%)	95 (63.3%)	
Rectum	324 (51.0%)	–	–	–	269 (55.5%)	55 (36.7%)	
Disease stage^6^				< **0.001**^b^			–
I	37 (6.3%)	10 (3.3%)	27 (9.6%)		–	–	
II	182 (31.0%)	110 (35.7%)	72 (25.7%)		–	–	
III	219 (37.2%)	93 (30.2%)	126 (45.0%)		–	–	
IV	150 (25.5%)	95 (30.8%)	55 (19.7%)		–	–	

Data presented as mean, standard deviation and absolute and relative numbers.

^a^Independent t student and ^b^Chi-squared tests performed. ^1^13 missing ^2^132 missing ^3^115 missing ^4^197 missing ^5^225 missing ^6^47 missing.

Significant values are in [bold].

Nutritional and BC characteristics of patients with cancer according to tumor site and presence of metastasis are shown in Table 2. Nearly half of the sample had a BMI within the normal range, with a small portion classified as underweight, and 44.8% were classified as overweight or obese. No significant differences were observed in patients’ BMI according to tumor localization; however, patients with metastasis had lower values of BMI (25.0 ± 4.6 kg/m^2^ vs. 24.0 ± 4.2 kg/m^2^, *p* = 0.016). Regarding BC, patients with rectal cancer had higher values for SAT, VAT and SMI compared to those with colon cancer. Patients with metastatic cancer had low SM and VAT when compared with those without metastasis.

**Table 2:** Nutritional and body composition characteristics of patients with cancer according to tumor type and metastasis (*N* = 635).

Characteristics	Total (*N* = 635)	Colon (*n* = 310)	Rectum (*n* = 324)	*p*	Without metastasis (*n* = 485)	With metastasis (*n* = 150)	*p*
Body mass index	24.7 ± 4.5	24.4 ± 4.3	25.1 ± 4.7	0.057^a^	25.0 ± 4.6	24.0 ± 4.2	**0.016**^a^
Underweight	41 (6.5%)	20 (6.5%)	21 (6.5%)	0.102^c^	29 (6.0%)	12 (8.0%)	0.137^c^
Normal range	310 (48.8%)	167 (53.9%)	143 (44.1%)		229 (47.2%)	81 (54.0%)	
Overweight	203 (32.0%)	88 (28.4%)	115 (35.5%)		158 (32.6%)	45 (30.0%)	
Obesity	81 (12.8%)	36 (11.6%)	45 (13.9%)		69 (14.2%)	12 (8.0%)	
Body composition							
SAT (cm^2^)	144.3 ± 82.2	142.9 ± 81.9	145.6 ± 82.6	0.680^a^	146.3 ± 85.2	137.8 ± 71.8	0.269^a^
VAT (cm^2^)	117.5 ± 79.7	115.7 ± 77.9	118.8 ± 81.7	0.630^a^	121.9 ± 83.0	102.5 ± 66.5	**0.004**^a^
IMAT (cm^2^)	10.2 ± 7.5	9.7 ± 7.0	10.7 ± 8.0	0.068^a^	10.5 ± 7.7	9.4 ± 6.8	0.137^a^
SM (cm^2^)	124.1 ± 32.9	120.4 ± 33.0	125.5 ± 32.9	< **0.001**^a^	124.4 ± 33.7	118.6 ± 30.3	**0.048**^a^
SMI (cm^2^/m^2^)	46.5 (39.5, 53.9)	45.2 (37.9, 52.0)	48.3 (41.0, 54.9)	< **0.001**^b^	46.9 (39.6, 54.2)	45.5 (39.1, 52.5)	0.223^b^
SMD (HU)	33.5 (27.1, 39.2)	33.9 (27.2, 39.2)	32.9 (27.1, 39.1)	0.546^b^	33.2 (26.9, 39.2)	33.8 (27.8, 38.9)	0.855^b^
Body composition abnormalities							
Low SMI	237 (37.3%)	135 (43.5%)	102 (31.5%)	**0.002**^c^	178 (36.7%)	59 (39.3%)	0.560^c^
Low SMD	436 (68.7%)	213 (68.7%)	223 (68.8%)	0.927^c^	327 (67.4%)	109 (72.7%)	0.226^c^
High VAT	286 (45.0%)	139 (44.8%)	147 (45.4%)	0.864^c^	229 (47.2%)	57 (38.0%)	**0.047**^c^

SAT, subcutaneous adipose tissue; VAT, visceral adipose tissue; IMAT, intermuscular adipose tissue; SM, skeletal muscle mass; SMI, skeletal muscle index; SMD, skeletal muscle radiodensity; UH, *Hounsfield Units*; BMI, body mass index. Data presented as median and interquartile range (P25, P75).

Low SMI: < 41 cm^2^/m^2^, regardless of BMI for women; < 43 cm^2^/m^2^ when BMI < 25 kg/m^2^ or < 53 cm^2^/m^2^ when BMI > 25 kg/m^2^; for men.

Low SMD: < 41 UH when BMI < 24.9 kg/m^2^ and < 33 UH when BMI > 25 kg/m^2^ for both sexes.

High VAT: 163.8cm^2^ for men and 80.1cm^2^ for women.

^a^Independent t student, ^b^Mann-Witney and ^c^Chi-squared tests performed.

Significant values are in [bold].

We also evaluated the associations between various tumor sites and presence of metastasis, as presented in Table 3. Patients with metastatic colon cancer had lower VAT and lower IMAT compared to those without metastasis. No statistical difference was found between BMI or BC measurements for patients with rectal cancer with and without metastasis.

**Table 3:** Nutritional and body composition characteristics of patients with cancer according to tumor type stratified by the presence of metastasis (*N* = 635).

Characteristics	Colon			Rectum		
	Without metastasis (*n* = 216)	With metastasis (*n* = 95)	*p*	Without metastasis (*n* = 269)	With metastasis (*n* = 55)	*p*
Body mass index	24.6 ± 4.5	23.8 ± 3.9	0.108^a^	25.2 ± 4.7	24.2 ± 4.7	0.148^a^
Underweight	14 (6.5%)	6 (6.3%)	0.288^c^	15 (5.6%)	6 (10.9%)	0.313^c^
Normal range	113 (52.3%)	54 (56.8%)		116 (43.1%)	27 (49.1%)	
Overweight	59 (27.3%)	29 (30.5%)		99 (36.8%)	16 (29.1%)	
Obesity	30 (13.9%)	6 (6.3%)		39 (14.5%)	6 (10.9%)	
Body composition						
SAT (cm^2^)	145.9 ± 86.7	136.1 ± 69.8	0.330^a^	146.6 ± 84.1	140.8 ± 75.7	0.633^a^
VAT (cm^2^)	122.6 ± 83.1	100.2 ± 62.2	**0.009**^a^	121.3 ± 83.1	106.6 ± 73.8	0.224^a^
IMAT (cm^2^)	10.2 ± 7.4	8.5 ± 5.7	**0.049**^a^	10.7 ± 8.0	11.0 ± 8.2	0.780^a^
SM (cm^2^)	121.5 ± 33.1	118.1 ± 32.8	0.408^a^	126.8 ± 34.0	119.5 ± 25.7	0.633^a^
SMI (cm^2^/m^2^)	45.8 (37.8, 52.2)	45.9 (38.0, 51.3)	0.947^b^	49.2 (40.9, 55.5)	47.3 (41.0, 53.7)	0.303^b^
SMD (HU)	33.2 (26.9, 39.3)	33.1 (28.0, 38.9)	0.916^b^	32.9 (26.9, 39.1)	32.8 (27.3, 39.2)	0.921^b^
Body composition abnormalities						
Low SMI	96 (44.4%)	39 (41.1%)	0.587^c^	82 (30.5%)	20 (36.4%)	0.392^c^
Low SMD	146 (67.6%)	67 (70.5%)	0.608^c^	181 (67.3%)	42 (76.4%)	0.185^c^
High VAT	105 (48.6%)	34 (35.8%)	**0.036**^c^	124 (46.1%)	23 (41.8%)	0.561^c^

SAT, subcutaneous adipose tissue; VAT, visceral adipose tissue; IMAT, intermuscular adipose tissue; SM, skeletal muscle mass; SMI, skeletal muscle index; SMD, skeletal muscle radiodensity; UH, *Hounsfield Units*; BMI, body mass index. Data presented as median and interquartile range (P25, P75).

Low SMI: < 41 cm^2^/m^2^, regardless of BMI for women; < 43 cm^2^/m^2^ when BMI < 25 kg/m^2^ or < 53 cm^2^/m^2^ when BMI > 25 kg/m^2^; for men.

Low SMD: < 41 UH when BMI < 24.9 kg/m^2^ and < 33 UH when BMI > 25 kg/m^2^ for both sexes.

High VAT: 163.8cm^2^ for men and 80.1cm^2^ for women.

^a^Independent t student, ^b^Mann-Witney and ^c^Chi-squared tests performed.

Significant values are in [bold].

To compare BC and other nutritional characteristics in patients without metastasis by tumor site, Table 4 displays BMI and BC comparisons of patients without metastasis according to tumor site. Notably, patients with colon cancer exhibited lower SMI compared to those with rectal cancer.

**Table 4:** Comparison of nutritional and body composition characteristics of patients with cancer without metastasis and according to tumor type (*n* = 485).

Characteristics	Colon without metastasis (*n* = 216)	Rectum without metastasis (*n* = 269)	*p*
Body mass index	24.6 ± 4.5	25.2 ± 4.7	0.161^a^
Underweight	14 (6.5%)	15 (5.6%)	0.130^c^
Normal range	113 (52.3%)	116 (43.1%)	
Overweight	59 (27.3%)	99 (36.8%)	
Obesity	30 (13.9%)	39 (14.5%)	
Body composition			
SAT (cm^2^)	145.9 ± 86.7	146.6 ± 84.1	0.931^a^
VAT (cm^2^)	122.6 ± 83.1	121.3 ± 83.1	0.866^a^
IMAT (cm^2^)	10.2 ± 7.4	10.7 ± 8.0	0.462^a^
SM (cm^2^)	121.5 ± 33.1	126.8 ± 34.0	0.085^a^
SMI (cm^2^/m^2^)	45.3 (37.8, 52.2)	48.6 (40.9, 55.5)	< **0.001**^b^
SMD (HU)	33.6 (26.9, 39.3)	33.0 (26.9, 39.1)	0.694^b^
Body composition abnormalities			
Low SMI	96 (44.4%)	82 (30.5%)	**0.002**^c^
Low SMD	146 (67.6%)	181 (67.3%)	0.943^c^
High VAT	105 (48.6%)	124 (46.1%)	0.581^c^

SAT, subcutaneous adipose tissue; VAT, visceral adipose tissue; IMAT, intermuscular adipose tissue; SM, skeletal muscle mass; SMI, skeletal muscle index; SMD, skeletal muscle radiodensity; UH, *Hounsfield Units*; BMI, body mass index. Data presented as median and interquartile range (P25, P75).

Low SMI: < 41 cm^2^/m^2^, regardless of BMI for women; < 43 cm^2^/m^2^ when BMI < 25 kg/m^2^ or < 53 cm^2^/m^2^ when BMI > 25 kg/m^2^; for men.

Low SMD: < 41 UH when BMI < 24.9 kg/m^2^ and < 33 UH when BMI > 25 kg/m^2^ for both sexes.

High VAT: 163.8cm^2^ for men and 80.1cm^2^ for women.

^a^Independent t student, ^b^Mann-Witney and ^c^Chi-squared tests performed.

Significant values are in [bold].

Regression models were used to assess the independent effect of tumor site and metastasis on BC. Collinearity was not detected among the variables included in the models, as confirmed by the variance inflation factor (VIF). Detailed information on collinearity diagnoses can be found in Supplementary Table 1. Model 1 adjusted for confounding factors like age, sex, and smoking status. Model 2 additionally included BMI. In Model 1, both tumor site and metastasis independently predicted changes in SM (cm^2^), showing an inverse association with SM for rectum tumors and metastasis. The model accounted for 54% of SMA variability (adjusted R^2^ = 0.54, *p* < 0.001). Metastasis alone was an independent predictor of changes in VAT (cm^2^) in Model 1, explaining 11% of VAT variability (adjusted R^2^ = 0.11, *p* = 0.016) with an inverse association (β − 17.4, 95% CI − 31.3 to − 3.6). In Model 2, with BMI included, tumor site emerged as the sole independent predictor of SMI changes, accounting for 50% of SMI variability (adjusted R^2^ = 0.50, *p* < 0.001). Notably, colon tumors were inversely associated with SM (β − 2.15, 95% CI − 3.3 to − 0.9). No other significant independent associations were observed (Table 5).

**Table 5:** Generalized linear-scale response regression: independent influence of tumor site and occurrence of metastasis on body composition of patients with colorectal cancer (*N* = 635).

Factors	SM (cm^2^)		SMI (cm^2^/m^2^)		SMD (HU)		IMAT (cm^2^)		VAT (cm^2^)	
	β (95% CI)	*p*	β (95% CI)	*p*	β (95% CI)	*p*	β (95% CI)	*p*	β (95% CI)	*p*
Model 1										
Tumor site										
Rectum	(Reference)	–	–	–	–	–	–	–	–	–
Colon	− 4.01 (− 7.5; − 0.4)	**0**.**026**	− 2.81 (− 4.2; − 1.4)	< **0**.**001**	0.05 (− 1.5; 1.0)	0.939	− 0.72 (− 1.8; 0.4)	0.204	1.24 (− 10.7; 13.2)	0.840
Metastasis										
No	(Reference)	–	–	–	–	–	–	–	–	–
Yes	− 4.18 (− 7.9; − 0.3)	**0**.**031**	− 0.50 (− 2.1; 1.1)	0.539	− 0.23 (− 1.5; 1.0)	0.734	− 0.73 (− 1.9; 0.5)	0.248	− 17.4 (− 31.3; − 3.6)	**0**.**014**
Model 2										
Tumor site										
Rectum	(Reference)	–	–	–	–	–	–	–	–	–
Colon	− 2.40 (− 1.5; 0.6)	0.122	− 2.15 (− 3.3; − 0.9)	< **0**.**001**	− 0.12 (− 1.3; 1.1)	0.847	− 0.33 (− 1.3; 0.7)	0.517	7.86 (− 1.6; 17.2)	0.104
Metastasis										
Without	(Reference)	–			–	–	–	–	–	–
With	− 1.81 (− 5.0; 1.4)	0.278	0.48 (− 0.8; 1.8)	0.496	− 0.48 (− 1.7; 0.8)	0.473	− 0.17 (− 1.3; 0.9)	0.776	− 7.75 (− 17.7; 2.2)	0.127
Model 3										
Tumor site										
Rectum	(Reference)	–	–	–	–	–	–	–	–	–
Colon	− 2.46 (− 6.5; 1.7)	0.250	− 2.22 (− 3.8; − 0.6)	**0**.**009**	0.39 (− 1.1; 1.9)	0.609	− 1.81 (− 3.1; − 0.5)	**0**.**008**	2.69 (− 10.7; 16.0)	0.694
Metastasis										
No	(Reference)	–	–	–	–	–	–	–	–	–
Yes	− 2.13 (− 6.5; 2.3)	0.346	0.29 (− 1.7; 2.3)	0.764	0.19 (− 1.2; 1.6)	0.800	− 0.49 (− 1.9; 0.9)	0.459	− 11.9 (− 25.1; 1.2)	0.076

Model 1: adjusted for age, sex, and smoking.

Model 2: adjusted for age, sex, smoking, and body mass index. Tumor site and presence of metastasis were incorporated within the same regression models, for better confounders control.

Model 3: adjusted for age, sex, BMI and all confounders found in DAGs, with the exception of income.

SAT, subcutaneous adipose tissue; VAT, visceral adipose tissue; IMAT, intermuscular adipose tissue; SM, skeletal muscle mass; SMI, skeletal muscle index; SMD, skeletal muscle radiodensity; UH, *Hounsfield Units*; BMI, body mass index; DAG, directed acyclic graphs.

Significant values are in [bold].

In the third regression model, which accounted for all potential confounders identified in the DAGs (Supplementary Figs. S1 and S2), we observed that the inverse association between tumor site (colon) and SMI persisted, despite the model exhibiting lower goodness-of-fit as indicated by higher AIC values. Patients with colon tumors had significantly lower SMI, compared to rectum tumors (β − 2.2, 95% CI − 3.8 to − 0.6). Moreover, in this model, we found that patients with colon tumors had significantly lower IMAT values (β − 1.8, 95% CI − 3.1 to − 0.5). A statistical trend was observed in which patients with metastasis tended to exhibit lower visceral adiposity.

## Discussion

Regression analyses indicated that, in a model not adjusted for BMI, both tumor site and metastasis independently influenced BC, affecting SM and visceral adiposity. However, when BMI is a confounding factor, many of these associations lost significance. These findings are noteworthy as they prompt a critical examination of whether tumor site and disease severity, such as the presence of metastasis, can genuinely account for changes in body composition. The results underscore the importance of considering body mass as a crucial confounder in such analyses. Lower body mass might naturally lead to reduced muscle mass and visceral adiposity. However, even after adjusting for BMI, tumor site (but not metastasis) independently explained SMI variations in our population.

There is an ongoing debate underscoring the crucial need to incorporate BMI as a standardization and/or adjustment measure when estimating BC, especially within populations with different body mass phenotypes. This approach is particularly relevant because individuals with varying body mass are prone to demonstrate distinct body composition profiles^16–18^. Therefore, our results emphasize the necessity of accounting for BMI to gain a more nuanced understanding of the relationship between tumor characteristics, disease severity, and alterations in BC. This nuanced perspective becomes pivotal in distinguishing the independent effects of tumor-related factors from those influenced by overall body mass.

Studies have reported an association between changes in adipose and muscle tissue with worse clinical outcomes in patients with CRC^19–21^. Low SM serves as a pivotal biomarker for unfavorable outcomes and constitutes a crucial component in the diagnosis of sarcopenia^22,23^. We observed that 37.3% of patients exhibited low low SMI. Nevertheless, it's crucial to acknowledge that this observation may be influenced by the demographic makeup of our study population, where over half of the participants were older adults. The decline in SM is an inherent aspect of the aging process^24^, contributing to the prevalence of low SM in our findings. Conversely, low SMD is an independent predictor of adverse events both in patients with cancer^25–27^ and in the aging population^28^. In our study, we found that 68.7% of patients with CRC had low SMD. Furthermore, a low SMD also points to worse general and CRC-specific mortality than those with a normal SMD^19,20,29^. It's noteworthy that the disparities observed in our study may have diverse impacts on prognosis. Supporting this hypothesis, previous research demonstrated that myosteatosis influenced the survival in patients with colon cancer but not in those with rectal cancer^19^, emphasizing the need to differentiate between the two when analyzing BC.

Although colon and rectal cancer are usually grouped together, there are several anatomical and mutagenic differences^10^. Hence, it is crucial to evaluate the specific impact on BC independently. The present study showed that patients with colon cancer had a more compromised profile of BC. The variations in disease staging may be explained by the distinct nature of rectal and colon cancers. Rectal cancer often manifests with well-defined symptoms, such as rectal bleeding with or without changes in bowel habits. In contrast, colon cancer-related symptoms are often vague in the early stages. Consequently, when the severity of symptoms require investigation, the disease is typically more advanced^30,31^. Therefore, these differences underscore the importance of distinct evaluations for colon and rectum as separate sites of cancer manifestation.

Similar to our findings, Xiao et al.^32^ also observed a higher prevalence of low SMI and low SMD in patients with colon cancer, compared to those with rectum cancer in a cohort of 3051 CRC patients, though their study included only stages I–III. Interestingly, our study, which included all cancer stages, including metastatic, found a higher frequency of metastasis in patients with colon cancer. This may partially explain the observed higher incidence of low SM in these patients. In turn, this reinforces the importance of distinguishing the anatomical site when considering BC assessments. In addition, similar to our crude analyses (see Table 2), some evidence have reported that patients with advanced CRC are more prone to BC alterations, leading to wasting conditions such as sarcopenia, cachexia, and malnutrition in comparison to patients with tumors in curative stages^33–35^, while high VAT seems to be related to earlier stage tumors and with a less aggressive tumor phenotype^36^.

Our study has several limitations to acknowledge. First, its design precludes observing changes in patient’s BC over time, making it impossible to determine cause-effect relationships between BC, cancer staging and tumor site. Second, other factors may be omitted from our linear regression models. Therefore, we recommend caution in interpreting and extrapolating our findings. The heterogeneity of our sample, including patients from various age groups, may limit the study’s external validity. However, this aspect mirrors the demographic profile of the Brazilian population affected by the disease^37^, which is a strength. Third, we did not account for differences in BC based on the location of colon cancer (right or left). Nevertheless, our study’s strength lies in its substantial sample size and inclusion of patients from various treatment centers across the country, enhancing its representativeness.

In conclusion, this study showed that patients with rectal cancer exhibited a higher SM, and significant BC differences exist in patients with CRC based on the presence or absence of metastases. For a more comprehensive understanding, future longitudinal studies are needed to track changes in BC in relation to other factors, such as the duration of the disease or types of treatments administered.

## Methods

### Study design and population

This study is part of a multi-center, retrospective cohort research involving patients from six Oncology Centers from Brazil, conducted between January 2016 and August 2022. For this cross-sectional analysis, data were collected from electronic medical records from six reference centers in cancer treatment in Brazil: Liga Norteriograndense Contra o Cancer (LIGA), Natal-RN; Onofre Lopes University Hospital (HUOL), Natal-RN; Ceará Cancer Institute (ICC), Fortaleza-CE; National Cancer Institute (INCA), Rio de Janeiro-RJ, State University of Campinas (Unicamp), Campinas-SP; and Federal University of Minas Gerais (UFMG), Belo Horizonte-MG. The study was performed in accordance with the Declaration of Helsinki^38^ and approved by the ethics committee of HUOL (protocol number 4.431.753). Due to the nature of the research, is exempted from informed consent.

Patients over 18 years of age, of both sexes, with a medical diagnosis of CRC between 2014 and 2017 were eligible to participate. Additionally, only patients who had undergone computed tomography (CT) imaging of the abdominal region within 90 days of the anthropometric evaluation were included. Patients were excluded from the study if their CT images did not include the third lumbar vertebra (L3), or if they had subcutaneous edema, ascites, or anatomical variations that could influence the analysis of BC by CT.

### Clinical and nutritional characteristics

Data were collected using a standardized form for all centers. Sociodemographic (sex, age, ethnicity, use of tobacco or alcohol intake, marriage status and educational level), anthropometric (weight and height), and clinical (cancer site and cancer staging) data were collected from electronic medical records. Anthropometric variables included weight and height and were used to calculate the body mass index (BMI), classified according to the WHO criteria^39^ regardless of age: underweight (< 18.5 kg/m^2^), normal weight (≥ 18.5 and < 25 kg/m^2^), overweight (≥ 25 and < 30 kg/m^2^) and obesity (≥ 30 kg/m^2^).

### Computed tomography image analysis

CT images retrieved from the electronic medical records of the hospitals were imported into the Radiant software for the selection of a single image (slice thickness of 1.2 mm) at the level of the L3. The selected images were analyzed using the Slice-O-Matic software (v.5, Tomovision), and the standard Hounsfield units (HU) established for each tissue were defined as follows: − 29 to 150 for SM, − 150 to − 50 for visceral adipose tissue (VAT) and − 190 to − 30 for intramuscular adipose tissue (IMAT) and subcutaneous adipose tissue (SAT)^40^.

Two trained researchers with anatomical expertise selected and analyzed the images (IMS and MCSM). To assess test–retest reliability, the interclass correlation coefficient (ICC) and the 95% confidence of interval (95% CI) were evaluated using 30 images based on single measurement, absolute-agreement, 2-way mixed-effects model. The ICC and CI for repeated measurements were 1.00 (1.00–1.00).

Alongside with SM, VAT, IMAT and SAT, we calculated the skeletal muscle index (SMI) (cm^2^/m^2^), which corresponds to the SMM corrected by the body surface. Additionally, we examined skeletal muscle radiodensity (SMD), which measures the mean radiation attenuation rate (HU). SMD is a radiological parameter used to evaluate intramuscular fat infiltration^14^.

Low SMI and low SMD (abnormal muscle “quantity” and “quality”) were defined according to the cutoff points proposed by Martin et al.^41^, as identified in a cohort study with adult patients diagnosed with gastrointestinal or lung cancer. For men, low SMI was defined as, SMI < 43 cm^2^/m^2^ when BMI < 25 kg/m^2^ or SMI < 53 cm^2^/m^2^ when BMI > 25 kg/m^2^. For women, low SMI was defined as, SMI < 41 cm^2^/m^2^, regardless of BMI. For low SMD, < 41 HU was adopted when BMI < 24.9 kg/m^2^ and < 33 UH when BMI > 25 kg/m^2^ for both sexes. High VAT was defined according to the cutoff point proposed by Doyle et al.^42^, which is 163.8 cm^2^ for men and 80.1 cm^2^ for women.

### Statistics analysis

Data were analyzed using SPSS version 22.0 statistical software. The normality of the sample was tested using the Kolmogorov–Smirnov test. Quantitative variables are presented as mean and standard deviation or median and interquartile range and compared using the t test for independent samples or the Mann–Whitney test, depending on the normality of the variables. Categorical variables are presented in absolute and relative numbers and compared using the Chi-square. Variables were tested for multicollinearity using the variance inflation factor (VIF > 0.10 < 3.0). Furthermore, directed acyclic graphs (DAGs) were constructed to identify all potential confounders pertaining to the independent and dependent variables. Subsequently, a generalized linear model with linear-scale response regression using a robust estimator was conducted to investigate the independent influence of tumor site and metastasis on body composition, while accounting for confounding variables. Models with adjustments were hierarchically constructed, and those with the best Akaike's information criterion (AIC) were included in the main text. A *p* value < 0.05 was considered statistically significant for all tests.

### Supplementary Information

Supplementary Information 1.

Supplementary Information 2.

Supplementary Information 3.

## Data Availability

The datasets generated during and/or analysed during the current study are not publicly available due to ethical and privacy restrictions but are available from the corresponding author on reasonable request.
