# Supplementary material for: Examining variations in body composition among patients with colorectal cancer according to site and disease stage
Source: Sci Rep. 2024 May 11;14:10829. doi: 10.1038/s41598-024-61790-0 (PMC11088614; doi:10.1038/s41598-024-61790-0)
Supplement: Supplementary file 1 — Supplementary Information 1. [file 41598_2024_61790_MOESM1_ESM.docx]

**Supplementary Table 1.** Variance Inflation Factor (VIF) coefficients for collinearity diagnostics.

| **Variables** | **SM (cm^2^)** | | **SMI (cm^2^/m^2^)** | | **SMD (HU)** | | **IMAT (cm^2^)** | | **VAT (cm^2^)** | |
| --- | --- | --- | --- | --- | --- | --- | --- | --- | --- | --- |
|  | **Tolerance** | **VIF** | **Tolerance** | **VIF** | **Tolerance** | **VIF** | **Tolerance** | **VIF** | **Tolerance** | **VIF** |
| Age | 0.98 | 1.02 | 0.98 | 1.02 | 0.98 | 1.02 | 0.98 | 1.02 | 0.98 | 1.02 |
| Sex | 0.97 | 1.03 | 0.97 | 1.03 | 0.97 | 1.03 | 0.97 | 1.03 | 0.97 | 1.03 |
| Educational level | 0.78 | 1.28 | 0.78 | 1.28 | 0.78 | 1.28 | 0.78 | 1.28 | 0.78 | 1.28 |
| Ethnicity | 0.98 | 1.02 | 0.98 | 1.02 | 0.98 | 1.02 | 0.98 | 1.02 | 0.98 | 1.02 |
| Smoking | 0.51 | 1.98 | 0.51 | 1.98 | 0.51 | 1.98 | 0.51 | 1.98 | 0.51 | 1.98 |
| Alcohol intake | 0.45 | 2.21 | 0.45 | 2.21 | 0.45 | 2.21 | 0.45 | 2.21 | 0.45 | 2.21 |
| TNM stage | 0.89 | 1.13 | 0.89 | 1.13 | 0.89 | 1.13 | 0.89 | 1.13 | 0.89 | 1.13 |
| Tumor site | 0.82 | 1.21 | 0.82 | 1.21 | 0.82 | 1.21 | 0.82 | 1.21 | 0.82 | 1.21 |
| Metastasis | 0.97 | 1.04 | 0.97 | 1.04 | 0.97 | 1.04 | 0.97 | 1.04 | 0.97 | 1.04 |
| BMI | 0.99 | 1.01 | 0.99 | 1.01 | 0.99 | 1.01 | 0.99 | 1.01 | 0.99 | 1.01 |

BMI: body mass index; IMAT: intermuscular adipose tissue; SM: skeletal muscle; SMD: skeletal muscle radiodensity; SMI: skeletal muscle index; TNM: tumor, node, metastasis classification; VAT: visceral adipose tissue. Values between tolerance and VIF (> 0.1 and < 3.0) were considered non-collinear.
