# Supplementary material for: Examining variations in body composition among patients with colorectal cancer according to site and disease stage
Source: Sci Rep. 2024 May 11;14:10829. doi: 10.1038/s41598-024-61790-0 (PMC11088614; doi:10.1038/s41598-024-61790-0)
Supplement: Supplementary file 3 — Supplementary Information 3. [file 41598_2024_61790_MOESM3_ESM.docx]

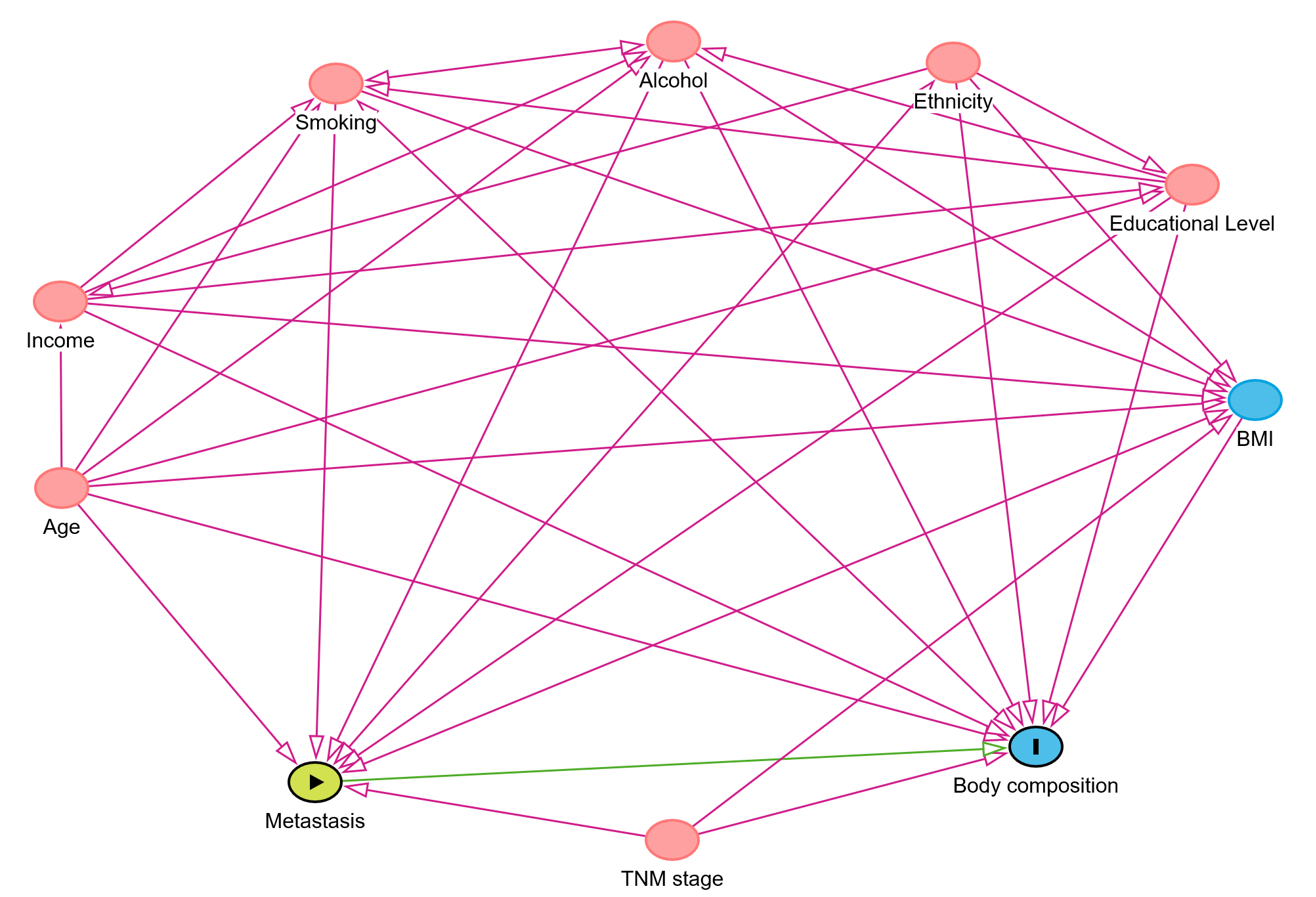


**Supplementary Figure 2.** Directed Acyclic Graphs for metastasis.

The green circle denotes the dependent variable, the blue circles indicate independent variables, and the pink circles represent confounder variables that are linked to both dependent and independent variables. Abbreviations: BMI, body mass index; TNM, tumor, node, metastasis classification.
